# Human Orthohantavirus Infections: A Narrative Review

**DOI:** 10.3390/pathogens15060652

**Published:** 2026-06-22

**Authors:** Vitor Duque

**Affiliations:** Faculty of Medicine (FMUC), University of Coimbra, 3004-504 Coimbra, Portugal; duque.vitor@gmail.com

**Keywords:** orthohantavirus, hantavirus, hemorrhagic fever with renal syndrome, hantavirus cardiopulmonary syndrome, zoonosis, endothelial dysfunction, emerging infectious diseases

## Abstract

Orthohantaviruses are zoonotic pathogens belonging to the family Hantaviridae and are responsible for significant human disease. These infections are characterized by acute systemic illness, vascular dysfunction, and, in severe cases, hemorrhage and multiorgan failure. Depending on the viral species involved, infection may result in hemorrhagic fever with renal syndrome (HFRS) or hantavirus cardiopulmonary syndrome (HCPS), both of which are associated with substantial morbidity and mortality. Rodents act as natural reservoirs, maintaining viral persistence in endemic ecosystems and enabling sporadic spillover to humans through exposure to infected excreta or contaminated environments. This review synthesizes current knowledge on rodent reservoir competence, hantavirus replication strategies, pathogenesis, clinical manifestations, ecological drivers of transmission, public health implications and future therapeutic developments and challenges. Understanding these mechanisms is essential for enhancing surveillance, risk assessment, and preventive strategies against orthohantavirus infections.

## 1. Introduction

Orthohantavirus infections are zoonotic viral diseases transmitted from rodents to humans and have likely been recognized for centuries. Historical descriptions consistent with clinical pictures likely to be of Hemorrhagic Fevers were documented in Chinese writings approximately 2000 years ago. However, international attention to these infections emerged during the Korean War (1950–1953), when more than 3000 United Nations troops developed “Korean hemorrhagic fever”, currently known as HFRS [1].

The etiological agent remained unidentified until the 1970s, when antigens detected predominantly in the lungs, but also in the parotid glands, liver, kidneys, and bladder of striped field mice (*Apodemus agrarius*) captured in South Korea, were shown to react with sera from patients with Korean hemorrhagic fever [2]. Experimental inoculation studies demonstrated viral antigen persistence in several organs, particularly the lungs, from 10 to 69 days after inoculation, although successful viral isolation was not initially achieved [2]. In addition, immunofluorescent antibody responses were detected in 97.4% of severe cases and 32.4% of milder clinically suspected cases. Antibodies became detectable within the first week after symptom onset, peaked during the second week, and persisted for up to 14 years [2].

Successful propagation of the virus in cell culture was finally achieved in 1981. The isolate, initially designated “KHF strain 76–118”, was later renamed Hantaan virus (HTNV) after the Hantan River, located near the Korean Demilitarized Zone, where the first recognized HFRS cases occurred [1,2].

In Europe, a milder clinical form of HFRS, termed nephropathia epidemica (NE), had already been recognized in Scandinavia since the 1930s. Its causative agent, Puumala virus (PUUV), was subsequently identified in bank voles (*Myodes glareolus*) in Finland during the early 1980s [3].

A major breakthrough in orthohantavirus research occurred in 1993 following an outbreak of severe acute respiratory illness in the Four Corners region of the southwestern United States, where Arizona, Colorado, New Mexico, and Utah intersect. This outbreak led to the recognition of a new clinical entity, hantavirus cardiopulmonary syndrome [3,4]. Comparative nucleotide sequence analyses demonstrated that the causative virus represented a novel orthohantavirus and established a direct genetic link between infected patients and rodent reservoir hosts [4]. The newly identified pathogen was later named Sin Nombre virus (SNV), and additional pathogenic hantaviruses, including Andes virus (ANDV), were subsequently identified throughout North and South America [3].

The genus orthohantavirus currently comprises 38 species for 60 distinct strains of recognized or potential public health relevance [5].

## 2. Epidemiology

Orthoantaviruses are emerging zoonotic pathogens responsible for HFRS in Eurasia and HCPS in the Americas.

Owing to their epidemic potential and associated morbidity and mortality, they have been classified by the Centers for Disease Control and Prevention as Category C priority pathogens [6].

Orthohantaviruses are traditionally divided into Old World and New World groups (Table 1) according to their geographic distribution and associated clinical syndromes [6].

More than 20 orthohantavirus species are currently recognized as pathogenic to humans, with transmission occurring predominantly through exposure to infected rodent reservoirs [6].

According to the latest European Centre for Disease Prevention and Control Annual Epidemiological Report (2023), 1885 orthohantavirus infections were reported across 28 European Union/European Economic Area countries, corresponding to an incidence of 0.4 cases per 100,000 population [7]. Cases occurred throughout the year, although incidence remained relatively low between January and March, increased progressively during spring, peaked in July and August, and subsequently declined through September [7]. Northern and Central European countries, particularly Finland, Germany, Sweden, Belgium, Austria, and France, continue to bear the highest disease burden [7]. However, Finland and Germany alone accounted for 60.5% of all reported cases.

Most infections occur in adults, with individuals aged 25 years and older accounting for 90.6% of cases. A predominance among males has also been observed, likely reflecting differences in occupational and environmental exposure [7]. Although mortality rates in Europe remain substantially lower than those observed in HCPS, severe disease and occasional fatalities continue to occur, particularly in infections caused by DOBV [7].

At the global level, approximately 200,000 orthohantavirus infections are estimated to occur annually, although the true burden is likely underestimated owing to asymptomatic or subclinical infections [6]. A recent meta-analysis estimated an overall global orthohantavirus seroprevalence of 2.93% (95% CI: 2.34–3.67), with regional differences observed across Asia, Europe, the Americas, and Africa [8]. Climatic factors also appear to influence orthohantavirus transmission dynamics. Progressively wetter early winters in northern regions have been associated with increased PUUV seroprevalence in bank vole populations and with a higher subsequent risk of human infection [9]. Population dynamics of bank voles further contribute to PUUV circulation, viral transmission, reinfection patterns, and the emergence of distinct viral lineages [3,6].

## 3. Rodents as Natural Reservoirs of Orthohantaviruses

These viruses are non-arthropod-borne viruses maintained primarily in rodents and insectivores, including shrews, moles, and bats [3,6].

Rodents constitute the principal natural reservoirs, supporting chronic infection and prolonged viral shedding without overt disease manifestations. Consequently, orthohantaviruses are classified as rodent-borne viruses (robovirus).

Although some investigations have demonstrated reduced survival, impaired growth, and histopathological alterations in infected reservoir hosts, persistent infection with minimal or absent clinical manifestations is generally considered the hallmark of natural host infection [6,7].

Orthoantaviruses exhibit marked host specificity, reflecting a long-standing co-evolutionary relationship between the virus and its natural reservoir [3,6]. Infections identified in other mammals, including moose, red foxes, domestic cats, and dogs, are generally interpreted as spillover events with limited epidemiological significance for human transmission [3].

The geographic distribution of orthohantaviruses closely parallels that of their reservoir hosts, supporting the hypothesis of millions of years of virus–host co-evolution [7].

The epidemiology of orthohantavirus infection is therefore strongly influenced by rodent population dynamics, climatic variability, environmental conditions, and human behaviour. Increased contact between humans and infected rodent reservoirs, particularly in rural, agricultural, and forested settings, remains the principal driver of zoonotic transmission [7,8].

Reservoir competence, species-specific virus associations, and ecological interactions are critical determinants of viral maintenance in nature and of human spillover risk.

## 4. Transmission Pathways and Spillover Dynamics

Human orthohantavirus infection is primarily acquired through inhalation of aerosolized rodent excreta, including urine, saliva, and feces, although transmission may also occur through direct contact with infected rodents or contaminated environments [3,6].

Chronically infected rodents shed the virus for extended periods, often over several months, with shedding levels varying according to the type of excreta. Although the environmental survival of orthohantaviruses has not been fully established, the infectious virus is thought to remain viable for up to two weeks under suitable environmental conditions [12].

The risk of human infection is strongly associated with environmental exposure, including land-cover characteristics, land-use changes, occupational activities, and recreational behaviors [8]. Most infections occur following inhalation of aerosolized viral particles originating from rodent saliva, urine, or feces [3], whereas infection through rodent bites is considered uncommon.

Human behaviors are a key determinant of hantavirus exposure and significantly influence the risk of infection. Both occupational and recreational activities that increase contact with rodent habitats have been associated with transmission. Identified risk activities include woodcutting, forestry and agricultural work, outdoor military training, camping, use of seasonal residences, visits to forest cabins, and entry into poorly ventilated buildings that may harbor rodent-contaminated dust.

Additional risk factors include rodent observation or trapping, residence in buildings with structural deficiencies that facilitate rodent entry, direct or indirect contact with rodents or their excreta, proximity to forested environments, and cigarette smoking. Furthermore, infections have been documented following exposure to wild rodents kept as pets or handled in research laboratory settings. In contrast to most orthohantaviruses, ANDV is unique in its demonstrated capacity for person-to-person transmission, particularly among individuals with close and prolonged exposure to infected patients [6]. Nevertheless, such transmission events remain exceptionally rare.

Prospective household-contact investigations conducted in Chile demonstrated that the risk of developing HCPS was substantially higher among sexual partners of index patients than among other household contacts (17.6% versus 1.2%, respectively) [25]. Risk factors independently associated with transmission included sexual contact, deep kissing, and sharing the same bed or room with an infected individual [25]. These findings strongly support the role of close interpersonal exposure in facilitating ANDV transmission.

Additional evidence for human-to-human transmission derives from the detection of viral RNA and proteins in multiple body fluids during acute infection [7,8,9]. ANDV genetic material and viral proteins have been identified in breast milk cells obtained from an infected mother who subsequently transmitted the infection to her child, suggesting that breastfeeding may represent a potential route of transmission through gastrointestinal exposure [7]. Although such transmission appears rare, these observations further distinguish ANDV from other orthoantaviruses and highlight the broad tissue distribution and shedding potential of the virus during acute disease [8].

The dynamics of viral shedding during ANDV infection were recently characterized in a prospective clinical study [8]. The highest proportion of ANDV-positive body fluid samples was observed during the first week after symptom onset, whereas positivity rates declined substantially by the third week [8]. During the acute phase, ANDV RNA was detectable in all buffy coat samples, remaining positive in 93% of patients between days 23 and 29 of illness [8]. Viral RNA was also identified in gingival crevicular fluid and saliva, although detection rates decreased over time, from 30% and 12% during the acute phase to 12% and 11%, respectively, during convalescence [8]. Importantly, infectious virus could be isolated from 42% of specimens collected during the acute phase, including saliva, gingival crevicular fluid, nasopharyngeal swabs, and urine [8].

These findings provide biologically plausible evidence supporting interpersonal transmission of ANDV and reinforce the importance of implementing infection prevention measures during the acute phase of illness, particularly among close household contacts and intimate partners [7,8,16]. Nevertheless, despite documented transmission events, the overall number of confirmed human-to-human cases remains limited when compared with the far greater burden of rodent-associated transmission [9].

### 4.1. Old World Orthohantaviruses

Old World orthohantaviruses are associated with HFRS in Europe and Asia and are maintained predominantly in rodent species belonging to the genera *Myodes*, *Microtus*, *Apodemus*, and *Rattus*, as well as in insectivores from the families Soricidae and Talpidae [3]. The most clinically relevant species include HTNV, PUUV, SEOV, and DOBV [3,6]. HTNV and SEOV predominate in Asia and are associated mainly with *Apodemus agrarius* and *Rattus norvegicus*, respectively. DOBV circulates primarily in the Balkans, Russia, and parts of Northern Europe and is considered the most virulent European orthohantavirus, with lineage-dependent differences in pathogenicity [3,6].

In Europe, PUUV is the predominant cause of nephropathia epidemica, a generally milder form of HFRS. The bank vole (*Myodes glareolus*), the principal reservoir host of PUUV, is the most widely distributed rodent reservoir species in Europe and northern Asia, contributing substantially to the extensive circulation of the virus [7]. Human seroprevalence and disease incidence correlate closely with infection prevalence in rodent populations, which demonstrates a marked north-to-south gradient across Europe, with lower prevalence in Mediterranean regions and higher rates in northern areas [7]. Reservoir abundance is influenced by complex multiannual and seasonal ecological fluctuations, including climatic conditions, food availability, and habitat characteristics [7,8].

Transmission among rodents occurs both directly, through biting and saliva exposure, and indirectly, via contaminated excreta [25]. Human infections are considered accidental events and are most frequently associated with occupational or outdoor exposure in endemic areas [3,25].

### 4.2. New World Orthohantaviruses

New World orthohantaviruses are responsible for HCPS throughout the Americas. Approximately 300 HCPS cases are reported annually, most occurring in Argentina, Chile, and Brazil [6]. Clinical severity varies according to the viral species involved. Severe disease and high mortality rates, ranging from 30% to 50%, are most frequently associated with SNV and ANDV, whereas milder clinical presentations have been described with CHOV and LNV [6]. Seasonal variation in HCPS incidence has also been observed, with peaks occurring during the austral spring and summer, coinciding with agricultural activities and ecological phenomena such as bamboo flowering that favor rodent population expansion [6,17].

Among all known hantaviruses, ANDV remains the only species for which person-to-person transmission has been convincingly documented [8,25]. Transmission appears to occur mainly among close household contacts or intimate partners following prolonged exposure to infected individuals, particularly through saliva or respiratory secretions [9]. Investigations of outbreaks in Argentina and Chile, including the large Epuyén outbreak (Chubut Province, Argentina), provided epidemiological and molecular evidence supporting interhuman transmission and documented up to four successive generations of infection [9,25]. Higher viral loads and more severe hepatic dysfunction were associated with transmission events during these outbreaks [9,25].

Nevertheless, important limitations remain regarding the interpretation of molecular epidemiological data. Identical viral sequences identified within the same endemic geographic region do not definitively prove person-to-person transmission, since exposure to a common environmental source cannot be excluded [25]. Although randomized controlled studies would theoretically provide definitive evidence, such investigations are ethically and practically unfeasible. Importantly, the absolute number of cases potentially attributable to interhuman transmission remains extremely limited compared with the substantially larger number of infections for which no evidence of secondary transmission has been observed, even in healthcare and household settings [25]. The 2026 outbreak of ANDV infection aboard the expedition cruise ship *MV Hondius* provided important epidemiological insights into the transmission dynamics of orthohantaviruses. A total of 13 cases (11 confirmed and 2 probable) were identified, including three fatalities, corresponding to a case-fatality rate of approximately 23%. The occurrence of infections in a confined setting involving passengers and crew from multiple countries highlighted the potential for international dissemination and underscored the challenges of outbreak investigation and control in highly mobile populations. Although the primary infections were presumed to have been acquired through exposure to infected rodent reservoirs in endemic areas of Argentina or Chile before embarkation, the temporal and epidemiological clustering of cases raised concerns regarding secondary transmission. These observations further supported previous evidence suggesting that close and prolonged contact with infected individuals may facilitate person-to-person spread of ANDV. Moreover, this event emphasized the importance of considering both travel history and epidemiological links when evaluating suspected cases, particularly in non-endemic regions [19].

## 5. Taxonomy and Replication Cycle

Orthohantaviruses, commonly referred to as hantaviruses (HTVs), belong to the order Bunyavirales, family Hantaviridae, subfamily Mammantavirinae, and genus Orthohantavirus [5,20]. The family Hantaviridae currently comprises at least seven genera and more than 50 viral species of recognized or potential public health importance [5]. Orthoantaviruses are important zoonotic pathogens responsible for HFRS in Eurasia and HCPS in the Americas.

Orthoantaviruses are enveloped RNA viruses with spherical to pleomorphic morphology, measuring approximately 80–120 nm in diameter and displaying characteristic spike-like surface projections [6]. Their genome consists of three single-stranded, negative-sense RNA segments designated as small (S), medium (M), and large (L), with approximate lengths of 1828, 3650, and 6550 nucleotides, respectively [6]. Overall genome size ranges from approximately 11,845 nucleotides in HTNV to 12,317 nucleotides in SNV [6]. The S segment encodes the nucleocapsid (N) protein, the M segment encodes the glycoprotein precursor (GPC), and the L segment encodes the viral RNA-dependent RNA polymerase (RdRp) [6]. Following translation, the GPC undergoes cleavage by host-cell signal peptidases, generating the two envelope glycoproteins Gn and Gc [6,20].

The envelope glycoproteins Gn and Gc assemble into spike complexes that mediate receptor attachment and membrane fusion during viral entry, with Gc playing the principal role in the membrane fusion process [21]. Orthoantaviruses infect endothelial, epithelial, and immune cells through interactions between viral glycoproteins and specific cellular receptors [3]. In addition to entry receptors, orthoantaviruses also exploit several host attachment factors that differ among viral species and contribute to viral tropism and pathogenicity [3]. Integrins, particularly β3-integrins, have been identified as important receptors for pathogenic hantaviruses, although no single universal receptor appears to be shared by all species [20,21]. Protocadherin-1 (PCDH1) acts as the primary host cell entry receptor for New World hantaviruses, including ANDV and SNV. Expression of PCDH1 on the surface of pulmonary endothelial cells facilitates viral attachment and entry, playing a critical role in the establishment of severe infection [22].

Following receptor binding, viral entry is initiated through glycoprotein-mediated attachment to the host cell surface and subsequent internalization via different endocytic pathways, depending on the viral species. These mechanisms include clathrin-mediated endocytosis, macropinocytosis, and clathrin-independent receptor-mediated endocytosis [20,21]. Once internalized, viral particles traffic through the endocytic pathway, where the acidic environment of endosomes induces conformational rearrangements in the Gc glycoprotein. This process exposes the fusion loop, enabling insertion into the endosomal membrane and fusion between viral and cellular membranes, ultimately releasing the viral nucleocapsid into the cytoplasm [17].

**Figure 1:**
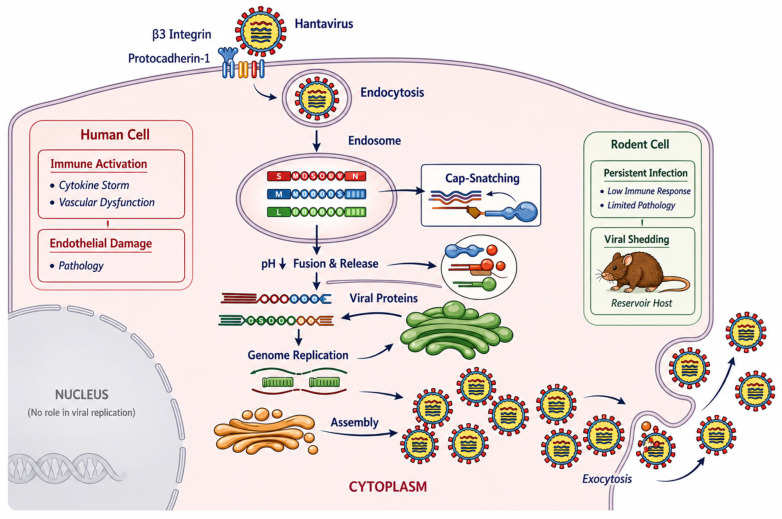
Schematic representation of the orthohantavirus replication cycle, highlighting virus entry, transcription, translation, assembly, release and pathological aspects in rodent vs. human hosts. Image obtained through artificial intelligence programming (ChatGPT, free version).

Viral transcription and genome replication occur in the cytoplasm and are mediated by the viral L protein [20,21]. During replication, orthohantavirus glycoproteins are directed to the Golgi apparatus, which serves as the principal site of viral assembly and budding for most orthohantavirus species [17]. Mature virions are subsequently transported to the plasma membrane and released by exocytosis (Figure 1).

Like many RNA viruses, orthohantaviruses exhibit considerable genetic variability, largely owing to the absence of proofreading and repair functions in the viral RNA-dependent RNA polymerase [20]. As a result, viral populations exist as genetically diverse quasispecies within their natural reservoir hosts. This diversity promotes rapid viral evolution and may confer selective advantages that enhance adaptation to host immune responses, ecological changes, and antiviral pressures [23].

Orthoantaviruses are relatively unstable in the environment compared with non-enveloped viruses. Like other enveloped viruses, they are readily inactivated by heat exposure, ultraviolet irradiation, organic solvents, detergents, and hypochlorite-containing disinfectants [6]. Exposure to 60 °C for 30 min is generally sufficient to inactivate infectious viral particles [6].

## 6. Pathogenesis

Orthohantavirus pathogenesis is a complex and multifactorial process driven by the interplay between viral replication, host immune responses, and endothelial dysfunction. Human infection typically occurs through inhalation of aerosolized viral particles derived from rodent urine, feces, or saliva present in contaminated environments [24,25]. Following entry into the host, orthoantaviruses primarily target vascular endothelial cells, although infection of epithelial cells, dendritic cells, and macrophages has also been described [24,25].

In contrast to their natural rodent reservoirs, in which persistent infection occurs with minimal or absent clinical manifestations due to effective immune modulation, orthohantavirus infection in humans triggers an intense and dysregulated immune response that substantially contributes to disease pathogenesis [25]. Viral replication within endothelial and immune cells induces activation of both innate and adaptive immune pathways, leading to the production of pro-inflammatory mediators and immune-mediated tissue injury [24,25,26].

A central component of hantavirus-associated pathology is the activation of virus-specific CD8+ cytotoxic T lymphocytes directed against infected endothelial cells. Although this response contributes to viral clearance, excessive immune activation simultaneously amplifies endothelial damage and vascular permeability [24,26]. Neutralizing antibodies develop early during the acute phase of infection and play an essential role in limiting viral dissemination and promoting viral clearance [26].

Marked cytokine and chemokine production further contributes to disease severity. Elevated circulating levels of tumor necrosis factor alpha (TNF-α), interleukin-6 (IL-6), interferon gamma (IFN-γ), and other inflammatory mediators promote a hyperinflammatory state that exacerbates endothelial activation and capillary leakage [25,26]. Importantly, orthoantaviruses generally do not produce direct cytopathic destruction of endothelial cells. Instead, endothelial dysfunction results predominantly from immune-mediated mechanisms that alter vascular barrier integrity [25,26].

Disruption of endothelial barrier function is considered the pathological hallmark of orthohantavirus disease. Increased vascular permeability leads to plasma extravasation, tissue edema, hypotension, and organ dysfunction [24,25]. In HCPS, capillary leakage predominantly affects the pulmonary microvasculature, resulting in rapidly progressive non-cardiogenic pulmonary edema, respiratory failure, and cardiogenic shock. In contrast, HFRS primarily involves renal microvascular dysfunction, manifesting as proteinuria, acute kidney injury, and hemorrhagic complications of varying severity [24].

Platelet dysfunction and abnormalities in coagulation pathways also contribute significantly to disease manifestations. Thrombocytopenia is a common finding in both HFRS and HCPS and may result from platelet consumption, endothelial activation, and altered platelet–endothelial interactions [24,25]. These alterations may further enhance vascular instability and contribute to hemorrhagic manifestations observed in severe disease.

Overall, current evidence indicates that orthohantavirus pathogenesis is largely mediated by endothelial infection, exaggerated inflammatory responses, and dysregulation of vascular permeability rather than by direct viral cytotoxicity [20,21,23]. The intensity and localization of the host immune response in specific target organs appear to be major determinants of clinical presentation and disease severity.

## 7. Clinical Manifestations

Human orthohantavirus infection manifests predominantly as two major clinical syndromes: HFRS, caused by Old World hantaviruses, and HCPS, caused by New World hantaviruses [3,24]. The principal Old World pathogenic species include HTNV, distributed mainly in Korea, China, and southeastern Russia, and DOBV, circulating predominantly in Eastern and southeastern Europe. In contrast, HCPS is primarily associated with SNV in North America and ANDV in Argentina and Chile, the latter representing the only orthohantavirus for which sustained person-to-person transmission has been convincingly documented [3,16].

The clinical divergence between HFRS and HCPS largely reflects differential involvement of specific vascular beds, with predominant injury of renal medullary capillaries in HFRS and pulmonary capillaries in HCPS [25,26]. Nevertheless, both syndromes share several pathogenic and clinical characteristics, including systemic endothelial dysfunction, increased vascular permeability, thrombocytopenia, hypotension, and leukocytosis with left shift [25,26]. The prodromal presentation is often similar across orthohantavirus infections and typically includes the abrupt onset of high fever, malaise, headache, myalgia, and influenza-like symptoms [24]. Overlapping manifestations are increasingly recognized, and both syndromes may exhibit renal and pulmonary involvement simultaneously. Respiratory manifestations, accompanied by radiographic abnormalities detectable on chest radiography or computed tomography, occur in nearly all patients with HCPS and in more than half of those with HFRS (Table 2).

### 7.1. Haemorrhagic Fever with Renal Syndrome

HFRS classically progresses through five sequential clinical phases: febrile, hypotensive, oliguric, polyuric (diuretic), and convalescent [3,25]. These stages are more clearly distinguishable in severe infections caused by HTNV and DOBV, whereas milder forms associated with PUUV or SEOV may show incomplete or overlapping phases [3]. Typical laboratory abnormalities include thrombocytopenia, elevated hematocrit, proteinuria, hypoalbuminemia, hematuria, elevated hepatic transaminases, and inflammatory marker elevation [3,25]. Following an incubation period of approximately 2–6 weeks, the disease usually begins abruptly with high fever, chills, headache, nausea, myalgia, abdominal pain, and lumbar pain [3]. Increased vascular permeability during the early febrile phase commonly leads to hypotension [3,24]. Neurological and ophthalmological manifestations are also frequent, including somnolence and visual disturbances, such as transient blurred vision and myopia resulting from lens thickening. Reduced visual acuity is a highly characteristic finding in the acute NE associated with PUUV infections. Overall, up to 70% of patients experience at least one ocular symptom [27].

Gastrointestinal manifestations and cutaneous rash may also occur, depending on the infecting viral strain [3].

The febrile phase generally persists for 3–7 days and is associated with thrombocytopenia, coagulation abnormalities, and hemorrhagic manifestations [3]. Subsequently, the hypotensive phase may last from several hours to two days. In severe cases, rapid hemodynamic deterioration may progress to irreversible shock, which accounts for approximately one-third of HFRS-related fatalities [3,24]. Thrombocytopenia and leukocytosis are particularly prominent during this stage. Hemorrhagic manifestations, considered markers of severe disease, may include conjunctival hemorrhages, petechiae, epistaxis, gastrointestinal bleeding, hematuria, metrorrhagia, and, rarely, fatal intracranial hemorrhage [3]. Severe bleeding complications occur more frequently in HTNV and DOBV infections, whereas PUUV infection is generally associated with milder hemorrhagic features [3,24].

The oliguric phase develops in approximately half of patients and usually persists for 3–7 days [3,24]. During this period, acute kidney injury becomes clinically evident, with oliguria or anuria, proteinuria, microscopic hematuria, azotemia, and abnormal urinary sediment [3]. Severe cases may require hemodialysis. Although blood pressure frequently normalizes during this phase, complications related to renal insufficiency, pulmonary edema, and hypertension may emerge [3]. Approximately half of HFRS-related deaths occur during the oliguric stage [3,24].

The subsequent polyuric phase is characterized by gradual recovery of renal function and increased urine output, often reaching several liters per day [3,24]. This phase may last from days to weeks and generally represents a favorable prognostic indicator [3]. Finally, the convalescent phase is marked by progressive normalization of clinical and laboratory abnormalities and may persist for up to six months [3]. Although complete recovery is common, some patients may develop long-term complications, including chronic renal dysfunction or persistent hypertension [3,24].

In children, the clinical manifestations of orthohantavirus infection are generally similar to those observed in adults but tend to be milder [3]. Pediatric patients with PUUV infection rarely require invasive medical interventions. Several studies have shown that the case-fatality is age-dependent. Overall, PUUV-associated nephropathia epidemica is associated with a mortality rate of approximately 0.4%, increasing to 6.5% among patients older than 80 years [28].

A retrospective analysis conducted in the United States over a 25-year period identified 719 cases of HPS. The vast majority of cases (94.6%) occurred in adults, whereas only 9.6% were reported in individuals younger than 18 years. The overall case-fatality rate was 35.4%, with no significant differences observed between pediatric and adult populations. However, the interval between symptom onset and death varied according to age. Children survived for a median of 2 days (interquartile range [IQR], 2–3 days), adolescents for 4 days (IQR, 3–5 days), and adults for 5 days (IQR, 4–8 days) following symptom onset (*p* = 0.001). In the United States, HPS is predominantly caused by SNV, which is associated with a mortality rate approaching 35% [29].

Evidence regarding orthohantavirus infection in immunocompromised patients remains limited. Although severe clinical manifestations have been reported, no definitive association between immunosuppression and increased mortality has been established. Further studies are required to clarify disease outcomes and optimal management in this population.

The clinical severity of HFRS varies considerably according to the infecting orthohantavirus species [3,24]. HTNV, AMRV, and DOBV infections are usually associated with severe disease and reported mortality rates between 5% and 15%, whereas SEOV infections typically produce moderate illness [3]. PUUV and SAAV infections are generally milder, with mortality rates below 1% [3]. However, substantial interindividual variability exists, and severe disease may occasionally occur even in PUUV infections [3]. The main causes of death include shock, acute renal failure, and multiorgan dysfunction [3,24].

### 7.2. Hantavirus Cardiopulmonary Syndrome

HCPS predominantly affects the respiratory and cardiovascular systems and is characterized by rapid progression and high mortality [25]. After an incubation period similar to that observed in HFRS, the disease begins with a nonspecific febrile prodrome lasting approximately 2–7 days [24]. Initial symptoms include fever, chills, headache, myalgia, arthralgia, retro-orbital pain, conjunctival injection, nausea, vomiting, abdominal pain, and diarrhea [3,24]. Abdominal pain may be sufficiently severe to mimic an acute surgical abdomen. Upper respiratory tract symptoms such as odynophagia and nasal congestion are generally absent during the early phase [24]. Some ANDV-infected patients may also develop petechiae involving the axillae and extremities [3,24].

In a subset of patients, the disease remains limited to the prodromal stage. However, in severe cases, abrupt progression to the cardiopulmonary phase occurs, characterized by cough, dyspnea, rapidly progressive non-cardiogenic pulmonary edema, respiratory failure, and frequently cardiogenic shock [24]. Increased pulmonary vascular permeability represents the principal pathogenic mechanism underlying respiratory compromise [24]. The cardiopulmonary phase generally lasts 2–4 days, and most fatalities occur within the first 24 h after hospital admission [24]. Survivors usually experience rapid recovery of endothelial barrier function following the acute phase [3,24].

Characteristic laboratory abnormalities include early thrombocytopenia, leukocytosis without toxic granulation, circulating immunoblasts, elevated hematocrit and hemoglobin levels, mild increases in serum creatinine and hepatic transaminases, lactate dehydrogenase elevation, hyponatremia, and proteinuria [24]. Thrombocytopenia has prognostic significance in both HCPS and HFRS and correlates with inflammatory severity and risk of acute kidney injury [3,24].

Radiological abnormalities evolve rapidly during the cardiopulmonary phase. Chest radiographs are often normal during the prodromal period but subsequently demonstrate bilateral interstitial and alveolar infiltrates, frequently accompanied by pleural effusions [24]. Chest CT commonly reveals interlobular septal thickening, ground-glass opacities, and pleural fluid accumulation [3,24].

HCPS remains associated with a high case-fatality rate, generally ranging from 30% to 50%, although early recognition and intensive supportive care significantly improve outcomes [3,6,24]. Severe disease occurs in more than half of SNV and ANDV infections, whereas CHOV infection is usually associated with a milder clinical course and less frequent progression to respiratory failure or shock [24].

## 8. Diagnosis

The diagnosis of HFRS and HCPS requires integration of clinical findings, epidemiological exposure history, and confirmatory laboratory testing [3,24]. Orthohantavirus infection should be suspected in patients presenting with acute febrile illness associated with headache, myalgia, abdominal or lumbar pain, and characteristic laboratory abnormalities, including thrombocytopenia, leukocytosis, elevated serum creatinine, proteinuria, and hematuria [3,24]. However, diagnosis based solely on clinical manifestations remains challenging, particularly in mild-to-moderate disease, because early symptoms are nonspecific and overlap substantially with those of other infectious syndromes [3].

A high index of clinical suspicion is warranted in individuals residing in or recently returning from endemic areas within the preceding 5–50 days who present with persistent fever lasting more than 48 h, headache, myalgia, gastrointestinal symptoms, and thrombocytopenia [6,24]. In more advanced stages, patients may develop acute kidney injury or respiratory compromise characterized by cough, dyspnea, hypoxemia, and bilateral pulmonary infiltrates [24]. In regions endemic for ANDV, close contact with an infected individual within the previous 40 days, particularly sexual contact or prolonged household exposure, should also be considered an important epidemiological risk factor because of the potential for person-to-person transmission [6,24]. Detection of proteinuria and hematuria by urine dipstick testing may provide an early diagnostic clue supporting suspicion of HFRS [3].

Because orthoantaviruses are potentially hazardous laboratory pathogens, biosafety precautions are required during specimen handling. Biosafety Level 2 (BSL-2) facilities and practices are recommended for laboratory manipulation of sera obtained from patients with suspected HCPS or HFRS [30].

### 8.1. Serological Diagnosis

Serological assays remain the cornerstone of orthohantavirus diagnosis and represent the most widely used laboratory methods worldwide [3,6,24]. The same diagnostic principles are generally applied for both HCPS and HFRS. Antibodies directed against the orthohantavirus nucleocapsid (N) protein are particularly useful because of their early appearance and strong immunogenicity [6,24].

Anti-orthohantavirus IgM antibodies usually become detectable at the onset of the febrile prodrome and indicate recent infection, although seroconversion may occasionally require up to two weeks [24]. IgG antibodies generally appear by the end of the febrile phase, persist lifelong in many patients, and are useful both for retrospective diagnosis and seroepidemiological studies [24]. The reference serological method for confirmation of acute infection remains enzyme-linked immunosorbent assay (ELISA/EIA) with both sensitivity and specificity approaching 95% [3,6,19,24].

Specific immunoglobulin M (IgM) and immunoglobulin IgG antibodies are typically detectable at the onset of symptoms. While IgG antibodies generally persist lifelong, IgM antibodies are primarily detected during the acute phase of infection and usually decline over a period of 2–6 months.

Rapid immunochromatographic IgM assays based on recombinant orthohantavirus nucleocapsid antigens are widely used for the diagnosis of PUUV-, HTNV-, and DOBV-associated HFRS [24]. These assays are available both as single-pathogen and multiplex detection formats and demonstrate diagnostic performance exceeding 90% compared with standard ELISA/EIA methods [6,24]. Certain PUUV-based immunochromatographic assays have also been applied for the detection of SNV and ANDV infections [24]. However, because cross-reactivity and false-positive reactions may occur, positive rapid-test results should ideally be confirmed using virus-specific ELISA/EIA assays [3,24].

IgG ELISA/EIA assays are commonly performed together with IgM testing during acute infection and are also extensively employed in seroprevalence and population-based studies [24]. Neutralization assays are not routinely used in clinical practice but remain important research tools for evaluating protective immunity and assessing candidate vaccines or monoclonal antibody therapies [24].

### 8.2. Molecular Diagnosis

Molecular methods have become increasingly important for the early diagnosis of orthohantavirus infection, particularly before seroconversion [6,24]. Commercial reverse transcription polymerase chain reaction (RT-PCR) and real-time quantitative RT-PCR (RT-qPCR) assays are currently available for the detection of orthoantaviruses associated with both HFRS and HCPS [6]. Most molecular assays target the S genomic segment and provide high sensitivity and specificity [6,24].

Nevertheless, PCR-based diagnostics have certain limitations. Because the viremic phase is generally restricted to the very early stages of infection, false-negative results may occur in low-viremic infections such as those caused by PUUV, or in patients tested later in disease [31].

Viral RNA loads are generally higher in the buffy coat than in plasma [24]. RT-qPCR has demonstrated the capacity to detect ANDV RNA up to two weeks before symptom onset and before the development of detectable antibody responses, as well as for several weeks after clinical recovery [24]. Consequently, molecular testing may allow earlier diagnosis and facilitate prompt identification of patients at risk of severe or fatal disease [3,24].

A sensitive internally controlled RT-qPCR assay targeting a consensus region in the N gene was developed for the specific detection and quantification of four hantavirus strains circulating in the Brazilian Amazon. This assay outperformed semi-nested RT-PCR, demonstrating high diagnostic accuracy (97.6%), clinical sensitivity (92.5%), and specificity (100%) [32].

Nested RT-PCR assays targeting the L segment have also demonstrated diagnostic utility [33]. Viral RNA may be detectable earlier in urine than in serum, and positivity in both specimen types can persist for up to one month after symptom onset [33]. These findings suggest that urine-based molecular testing may represent a useful complementary diagnostic approach, particularly during the early stages of infection [33].

## 9. Treatment

No specific antiviral or immunomodulatory therapy has been proven effective; consequently, management of hospitalized patients remains predominantly supportive. Intravenous ribavirin has not demonstrated a survival benefit in HFRS or during the cardiopulmonary phase of HCPS [24,34].

Management of HFRS relies on close clinical monitoring, including vital signs, blood pressure, urine output, and fluid and electrolyte balance. Supportive care includes analgesics for pain relief, blood transfusions, intravenous fluids for hypotension, oxygen therapy for hypoxia, and correction of electrolyte imbalances.

Patients with HCPS are infrequently hospitalized during the prodromal phase and may rapidly progress to severe cardiopulmonary compromise, shock, and death before confirmatory serological or molecular test results become available. Consequently, decisions regarding early transfer to centers with extracorporeal membrane oxygenation (ECMO) capability, as well as the timely establishment of vascular access for potential ECMO support, should be guided primarily by clinical suspicion and characteristic findings from routine laboratory investigations rather than laboratory confirmation alone [3,24,34].

During the cardiopulmonary phase, a presumptive diagnosis of HCPS can be supported by characteristic hematological findings identified on routine blood counts and peripheral blood smears. The presence of at least four of the following five criteria—thrombocytopenia, a left shift in the granulocytic lineage, absence of toxic granulation in myeloid cells, hemoconcentration, and immunoblasts accounting for more than 10% of circulating leukocytes—has been reported to yield a sensitivity of 96% and a specificity of 99% for the diagnosis of HCPS [3,24].

Several routinely available laboratory parameters have demonstrated prognostic value in HCPS. A platelet count exceeding 115,000/μL at hospital admission has been associated with a reduced likelihood of progression to severe disease, whereas severe thrombocytopenia, particularly platelet counts below 40,000/μL, has been linked to an increased risk of mortality [3,24,34].

In addition, the presence of significant proteinuria on admission has been identified as an adverse prognostic marker and has been associated with a higher risk of fatal outcomes. Once clinical or laboratory indicators of circulatory shock emerge, continuous hemodynamic monitoring is essential to enable the timely initiation of advanced organ support therapies. Patients with HCPS may deteriorate rapidly, progressing within hours from minimal oxygen requirements to respiratory failure requiring invasive mechanical ventilation, and from stable hemodynamic status to refractory shock [3]. In cases where cardiogenic shock develops despite aggressive inotropic support and severe respiratory compromise persists despite optimal mechanical ventilation, ECMO should be considered as a rescue therapy, particularly in patients with refractory cardiopulmonary failure [24,34].

The pathophysiology of HCPS is primarily characterized by increased pulmonary capillary permeability, resulting in non-cardiogenic pulmonary edema and profound intravascular fluid loss. As the disease progresses, patients may develop reduced preload and worsening hypovolemia, accompanied by impaired cardiac output and, in severe cases, a marked increase in systemic vascular resistance. These hemodynamic abnormalities contribute to the rapid onset of cardiopulmonary failure and are key determinants of disease severity and clinical outcome [24].

The presence of clinical signs of tissue hypoperfusion, such as skin mottling and prolonged capillary refill time, or evidence of hyperlactatemia, should prompt early initiation of hemodynamic monitoring and appropriate titration of inotropic support. Assessment of cardiac index is particularly important in patients with suspected or evolving circulatory failure and can be achieved using thermodilution-based methods, including transpulmonary thermodilution or pulmonary artery catheterization. In settings with continuous access to experienced personnel, serial echocardiographic evaluation represents a less invasive alternative for monitoring cardiac function and tracking dynamic changes in cardiac index over time [24,34].

Volume resuscitation should be approached with caution in patients with HCPS, as excessive fluid administration may exacerbate pulmonary edema and has been associated with worse clinical outcomes. Instead, optimization of cardiac output through inotropic support remains the cornerstone of hemodynamic management [24].

Although supplemental oxygen and ventilatory support are frequently required, endotracheal intubation should be deferred whenever clinically feasible until vascular access for potential ECMO support has been secured. In patients who develop refractory shock despite treatment with inotropic agents, such as dobutamine or epinephrine, or who fail to achieve adequate gas exchange with conventional respiratory support, early initiation of venoarterial extracorporeal membrane oxygenation (VA-ECMO) should be strongly considered [24,34].

## 10. Prevention

Preventive strategies against orthohantavirus infection are primarily aimed at reducing human exposure to infected rodents and their excreta, particularly urine, feces, and saliva, which constitute the principal routes of transmission [34]. Environmental control measures remain the cornerstone of prevention in endemic regions and include rodent-proofing of homes and storage facilities, safe food storage, elimination of rodent nesting sites, and appropriate waste management [7,34,35]. Public health recommendations also emphasize adequate ventilation of enclosed spaces before cleaning and advise against sweeping or vacuuming potentially contaminated environments, as these activities may aerosolize infectious viral particles [7,34,35]. Instead, contaminated surfaces should be disinfected using bleach-based or other approved disinfectant solutions while gloves and, when appropriate, respiratory protection are used [34].

Occupational exposure represents an important risk factor for orthohantavirus infection. Agricultural workers, forestry personnel, military staff, pest-control workers, and laboratory professionals should adopt personal protective measures, including gloves, protective clothing, and respiratory protection in high-risk settings [34,35]. Public health education campaigns directed toward populations living in endemic areas are essential to improve awareness regarding transmission routes, rodent exposure, and preventive behaviors [35]. In addition, surveillance systems integrating ecological monitoring of rodent populations and climatic or environmental factors may contribute to early risk assessment and outbreak prevention [10,11,35].

In the Americas, particularly in regions where ANDV circulates, limited person-to-person transmission has been documented [10,11]. Consequently, infection prevention and control strategies should include prompt identification and isolation of suspected cases, contact tracing, and implementation of droplet and contact precautions in healthcare settings [21,34]. Standard and droplet precautions are generally recommended for the care of symptomatic individuals, with escalation to airborne precautions when aerosol-generating procedures are performed [34]. Although nosocomial transmission appears uncommon, hospital-associated cases have been described during outbreaks, reinforcing the importance of strict adherence to infection-control practices [19]. Asymptomatic individuals classified as high-risk contacts should undergo active surveillance and remain quarantined (at home- or facility-based) for 42 days after their most recent exposure. These individuals should perform passive self-monitoring and seek medical attention promptly if compatible symptoms emerge.

For passengers and crew members in confined settings, such as cruise ships or other outbreak-associated environments, enhanced preventive measures are advisable, including frequent hand hygiene, respiratory etiquette, physical distancing when feasible, and close monitoring for compatible symptoms [19].

At present, no universally approved vaccine is available for the prevention of orthohantavirus infection, although several vaccine candidates, monoclonal antibodies, and immunotherapeutic strategies remain under investigation [3,24]. Therefore, current preventive strategies continue to rely predominantly on rodent control, environmental hygiene, occupational protection, early case recognition, and public health education.

## 11. Future Therapeutic Developments and Challenges

Although considerable progress has been made in elucidating the biology, epidemiology, and clinical features of orthohantaviruses, important gaps in knowledge persist.

Future research should focus on elucidating the molecular mechanisms underlying viral pathogenesis, host susceptibility, and the determinants of disease severity and person-to-person transmission, particularly for the Andes virus.

Enhanced ecological and genomic surveillance is needed to improve the detection of emerging orthohantaviruses and to better understand virus–host interactions and geographic spread.

In addition, the development of rapid, standardized diagnostic tools, effective antiviral therapies, and broadly protective vaccines remains a major priority.

No universally approved specific antiviral treatment is currently available for hantavirus infections. Consequently, considerable research efforts are focused on developing novel therapeutic approaches targeting both viral replication and host-mediated pathogenic mechanisms. Recent advances in structural virology have improved the understanding of critical stages of the hantavirus replication cycle, facilitating the identification of novel therapeutic targets [20].

Several antiviral compounds have demonstrated promising activity in experimental models. Viral entry inhibitors, including griffithsin, lactoferrin, coumarin derivatives, fusion-inhibiting peptides, and receptor-targeting cyclic nonapeptides, aim to prevent viral attachment and cellular entry. In parallel, direct-acting antivirals targeting viral replication, such as ribavirin, ETAR, favipiravir, baloxavir acid, and small interfering RNA (siRNA)-based therapies, have shown varying degrees of efficacy in vitro and in animal models [36,37]. However, the clinical benefits of these agents remain uncertain, largely due to limited human studies, variability among orthohantavirus species, and the narrow therapeutic window associated with the rapid progression of severe disease [3,22].

Growing evidence suggests that disease severity is driven not only by viral replication but also by dysregulated host immune responses. Consequently, host-directed therapeutic strategies have emerged as attractive alternatives or adjuncts to conventional antivirals. Agents such as vandetanib and bradykinin B2 receptor antagonists have demonstrated the potential to reduce vascular leakage and endothelial dysfunction, hallmarks of severe hantavirus disease [37]. Furthermore, recent studies have highlighted the ability of orthohantaviruses to evade innate immune responses in reservoir hosts, providing further rationale for the development of immunomodulatory therapeutic approaches [26].

Passive immunotherapy represents another promising avenue. Neutralizing antibodies (NAbs), administered either as monoclonal or polyclonal preparations, have shown protective effects in preclinical studies and may offer therapeutic benefits when administered early in infection [37,38]. Advances in structural biology and antibody engineering have facilitated the identification of highly potent broadly neutralizing antibodies targeting conserved glycoprotein epitopes across multiple hantavirus species [37]. Such approaches could provide both post-exposure prophylaxis and therapeutic intervention during outbreaks caused by highly pathogenic orthohantaviruses [37,38].

Vaccine development has also accelerated considerably in recent years. Multiple platforms, including recombinant protein vaccines, viral vector-based vaccines, DNA vaccines, and mRNA-based technologies, are being evaluated in preclinical and clinical studies [38]. Recent investigations of glycoprotein-based nucleic acid vaccines have demonstrated encouraging immunogenicity and protective efficacy, highlighting the potential of next-generation vaccine platforms for broad hantavirus prevention [38]. Nevertheless, no vaccine has yet achieved widespread global implementation, and further studies are needed to establish long-term protection and cross-reactive immunity against genetically diverse orthohantaviruses [38,39].

A major priority for future research is the characterization of the cytokine and chemokine networks involved in disease pathogenesis. Excessive production of pro-inflammatory mediators contributes to endothelial dysfunction, increased vascular permeability, and organ injury. Improved understanding of these mechanisms may facilitate the development of targeted immunotherapies capable of modulating harmful inflammatory responses while preserving antiviral immunity [26,36]. Furthermore, the identification of reliable biomarkers associated with disease severity could enable earlier risk stratification and more personalized therapeutic interventions.

Despite these promising developments, several challenges remain. The rarity and geographic clustering of many hantavirus infections complicate the design of adequately powered clinical trials. In addition, substantial genetic diversity among orthohantaviruses may limit the effectiveness of species-specific interventions. The lack of standardized animal models and incomplete understanding of correlates of protection further hinder therapeutic and vaccine development [36,39]. Addressing these limitations will require coordinated international research efforts, enhanced surveillance systems, and collaborative clinical networks capable of evaluating emerging therapeutic candidates.

Overall, the future management of hantavirus infections will likely rely on a multifaceted approach combining direct-acting antivirals, host-targeted therapies, passive immunization, and effective vaccines. Continued advances in molecular virology, immunology, structural biology, and translational research are expected to accelerate the development of safe and effective interventions against these globally important zoonotic pathogens.

## 12. Conclusions

Orthohantaviruses remain important emerging zoonotic pathogens of global public health relevance, responsible for significant morbidity and mortality across both endemic and non-endemic regions. Although traditionally classified into Old World and New World groups associated with HFRS and HCPS, respectively, increasing evidence demonstrates substantial overlap in clinical manifestations, pathophysiological mechanisms, and host immune responses.

Advances in molecular virology, epidemiology, and immunopathogenesis have considerably improved the current understanding of orthohantavirus infection. Nevertheless, important gaps remain regarding the determinants of disease severity, viral persistence in reservoir hosts, mechanisms of endothelial injury, and the precise contribution of host immune responses to clinical outcomes. Climatic and ecological changes influencing rodent population dynamics are likely contributing to the expanding geographic distribution and emergence of orthohantavirus infections worldwide, reinforcing the need for integrated One Health surveillance strategies.

The diagnosis of orthohantavirus infection continues to rely on a combination of epidemiological suspicion, characteristic clinical findings, and laboratory confirmation through serological and molecular methods. Early recognition remains challenging because of the nonspecific nature of the prodromal phase, yet it is essential to improve clinical outcomes, particularly in HCPS, where rapid cardiopulmonary deterioration may occur.

Current management remains predominantly supportive, with intensive care interventions frequently required in severe cases. Despite ongoing research into antiviral therapies, monoclonal antibodies, and immunomodulatory approaches, no universally effective specific treatment has yet been established. Likewise, no globally approved vaccine is currently available, and prevention continues to depend largely on rodent control, environmental hygiene, occupational protection, public health education, and outbreak surveillance.

The unique capacity of Andes virus for person-to-person transmission further highlights the complexity of orthohantavirus epidemiology and emphasizes the importance of infection prevention and control measures in healthcare and community settings. Recent evidence demonstrating viral shedding in multiple body fluids and possible transmission through close interpersonal contact underscores the necessity for continued vigilance and further investigation into transmission dynamics.

Future research should prioritize the development of rapid diagnostic tools, effective vaccines, targeted antiviral therapies, and a deeper understanding of host–virus interactions. Strengthening international surveillance networks and multidisciplinary collaboration will be essential to anticipate emerging outbreaks and mitigate the global burden of orthohantavirus disease.

## Figures and Tables

**Table 1:** Old and new world orthohantavirus species, reservoirs, geographic distribution and human disease [3,4,5,6,7,8,9,10,11,12,13,14,15,16,17,18,19,20,21,22,23,24].

Virus Species	Geographic Distribution	Principal Reservoir Host	Human Disease
**Old World Orthohantaviruses**			
Hantaan virus (HTNV)	China, Russia, Korea	*Apodemus agrarius*	Severe HFRS
Amur virus (AMRV)	Eastern Russia, Northeastern China	*Apodemus peninsulae*	HFRS
Seoul virus (SEOV)	Worldwide	*Rattus norvegicus*, *R. rattus*	HFRS
Puumala virus (PUUV)	Northern/Central Europe	*Myodes glareolus*	Nephropathia epidemica (mild HFRS)
Dobrava-Belgrade virus (DOBV)	Balkans/Central/Eastern Europe	*Apodemus flavicollis*	HFRS
Saaremaa virus (SAAV)	Baltic/Eastern Europe	*Apodemus agrarius*	HFRS
Tula virus (TULV)	Central/Eastern Europe	*Microtus arvalis*	Rare
**New World Orthohantaviruses**			
Sin Nombre virus (SNV)	USA, Canada	*Peromyscus maniculatus*	HCPS
New York virus (NYV)	Northeastern USA	*Peromyscus leucopus*	HCPS
Monongahela virus (MGLV)	Eastern USA	*Peromyscus maniculatus*	HCPS
Bayou virus (BAYV)	Southern USA	*Oryzomys palustris*	HCPS
Black Creek Canal virus (BCCV)	Southeastern USA	*Sigmodon hispidus*	HCPS
Choclo virus (CHOV)	Panama	*Oligoryzomys fulvescens*	HCPS
Andes virus (ANDV)	Argentina, Chile	*Oligoryzomys longicaudatus*	HCPS
Laguna Negra virus (LNV)	Paraguay, Bolivia	*Calomys laucha*	HCPS
Juquitiba virus (JUQV)	Brazil, Paraguay, Uruguay	*Oligoryzomys nigripes*	HCPS
Araraquara virus (ARAV)	Brazil	*Necromys lasiurus*	HCPS
Castelo dos Sonhos virus (CASV)	Brazil	*Oligoryzomys* spp.	HCPS
Rio Mamoré virus (RIOMV)	Bolivia, Peru	*Oligoryzomys microtis*	HCPS

**Table 2:** Comparative clinical features of HFRS and HCPS [3,24].

Feature	HFRS	HCPS
Prodromal Symptoms	Fever, chills, headache, myalgia, nausea, vomiting	Fever, chills, headache, nausea, vomiting, diarrhea
Renal Involvement	Acute kidney injury, oliguria, proteinuria, hematuria	Mild renal involvement
Pulmonary Involvement	Rare, mild	Pulmonary edema, respiratory failure, hypoxia
Cardiovascular Manifestations	Hypotension, shock in severe cases	Hypotension, shock, cardiac compromise
Hemorrhagic Manifestations	Petechiae, ecchymoses, mild bleeding	Rare, minor bleeding
Laboratory Findings	Thrombocytopenia, elevated hematocrit and transaminases, proteinuria	Thrombocytopenia, elevated hematocrit, LDH, transaminases, leukocytosis
Mortality Rate	1–15% (higher for HTNV/DOBV, lower for PUUV)	30–50% (may exceed 50% in severe cases)
Person-to-Person Transmission	Not reported	Only for ANDV
Long-term Sequelae	Full recovery, occasional residual renal impairment	Possible long-term pulmonary/cardiac sequelae

## Data Availability

No new data were created or analyzed in this study. Data sharing is not applicable to this article.
